# Performance Assessment of Heartbeat Detection Algorithms on Photoplethysmograph and Functional NearInfrared Spectroscopy Signals

**DOI:** 10.3390/s23073668

**Published:** 2023-03-31

**Authors:** Andrea Bizzego, Gianluca Esposito

**Affiliations:** Department of Psychology and Cognitive Science, University of Trento, 38068 Rovereto, Italy; gianluca.esposito@unitn.it

**Keywords:** heartbeat detection, wearable devices, photoplethysmography, sensors

## Abstract

With wearable sensors, the acquisition of physiological signals has become affordable and feasible in everyday life. Specifically, Photoplethysmography (PPG), being a low-cost and highly portable technology, has attracted notable interest for measuring and diagnosing cardiac activity, one of the most important physiological and autonomic indicators. In addition to the technological development, several specific signal-processing algorithms have been designed to enable reliable detection of heartbeats and cope with the lower quality of the signals. In this study, we compare three heartbeat detection algorithms: Derivative-Based Detection (DBD), Recursive Combinatorial Optimization (RCO), and Multi-Scale Peak and Trough Detection (MSPTD). In particular, we considered signals from two datasets, namely, the PPG-DALIA dataset (N = 15) and the FANTASIA dataset (N = 20) which differ in terms of signal characteristics (sampling frequency and length) and type of acquisition devices (wearable and medical-grade). The comparison is performed both in terms of heartbeat detection performance and computational workload required to execute the algorithms. Finally, we explore the applicability of these algorithms on the cardiac component obtained from functional Near InfraRed Spectroscopy signals (fNIRS).The results indicate that, while the MSPTD algorithm achieves a higher F1 score in cases that involve body movements, such as cycling (MSPTD: Mean = 74.7, SD = 14.4; DBD: Mean = 54.4, SD = 21.0; DBD + RCO: Mean = 49.5, SD = 22.9) and walking up and down the stairs (MSPTD: Mean = 62.9, SD = 12.2; DBD: Mean = 50.5, SD = 11.9; DBD + RCO: Mean = 45.0, SD = 14.0), for all other activities the three algorithms perform similarly. In terms of computational complexity, the computation time of the MSPTD algorithm appears to grow exponentially with the signal sampling frequency, thus requiring longer computation times in the case of high-sampling frequency signals, where the usage of the DBD and RCO algorithms might be preferable. All three algorithms appear to be appropriate candidates for exploring the applicability of heartbeat detection on fNIRS data.

## 1. Introduction

The diffusion of wearable devices (in short: “wearables”) enables the monitoring of physiological signals in numerous real-life contexts. While the reduced restriction of body movements that wearable technology allows is one key advantage, it also presents an issue regarding the quality of the signals, i.e., Motion Artifacts (MA).

The main sensing technology employed to measure cardiac activity with wearables is based on photoplethysmography (PPG) [1]. Signals collected by means of PPG to measure the cardiac activity are called Blood Volume Pulse (BVP) signals. To deal with MA, several algorithms and technical solutions have been designed (see for instance [2,3,4] for a review). However, due to the impulsive and time-dependent nature of body movements, MA removal presents a challenging task.

Alternative approaches rely on the rejection of signals with low-signal quality [3,5]. However, in addition to reducing the available data and applicability, these approaches additionally require ensuring the reliability of the rejection algorithms.

To compare the performance of the different algorithms, datasets have also been made available. For instance, the Wearable and Clinical Signals dataset [6] includes signals from wearables and medical-grade devices, PhysioNet [7] provides datasets of cardiac signals with annotated heartbeats, the PPG-DALIA dataset includes BVP signals collected with wearables during various daily activities [8].

Based on the PPG-DALIA dataset, Charlton and colleagues [9] performed an accurate comparison of the most used algorithms for heartbeat detection on BVP signals. Specifically, they evaluated the performance of the algorithms on eight different activities, acquired from 15 subjects. Among the 15 algorithms analyzed, the Multi-Scale Peak and Trough Detection (MSPTD) algorithm [10] demonstrated the best performance, together with the Adapted Onset Detector algorithms [11].

While the study provides a valuable reference for the state-of-the-art algorithms for heartbeat detection in BVP signals, there are some critical aspects that were not included and require additional investigation. First, the review did not consider two algorithms, the Derivative-based Beat Detection (DBD) and the Recursive Combinatorial Optimization (RCO). Another study that introduced the two algorithms [12] focused mainly on the description of the algorithms and the assessment of their validity for the extraction of Heart Rate Variability indicators, not on the comparison of the heartbeat detection performance with existing algorithms.

Second, the procedure adopted in the study of Charlton and colleagues [9] included a preliminary step to reject poor-quality signals, and an assessment of the heartbeat detection performance was based only on good-quality signals. This initial assessment was needed to provide an indication of the maximal performance that can be expected; however, it is also important to investigate the performance on real-life data, where compliance with high-quality standards is not always satisfied.

Third, once the validity of an algorithm has been established, it is important to also consider the technical aspects that can affect its usability and applicability, one of the most important factors being the computational complexity. Algorithms that involve intensive computational steps might be more appropriate for research applications, while real-life contexts, such as health monitoring or biofeedback, require algorithms with reduced computation times. Since the computational complexity depends on the cardinality of input data, an assessment of the computational complexity for signal-processing algorithms needs to include an investigation of the effects of the duration and sampling frequency of the signals.

In this paper, we aim at extending the review of Charlton and colleagues [9] by: (a) comparing the DBD and RCO algorithms with the MSPTD algorithm on signals of unknown quality, from wearable and medical-grade devices; and (b) assessing their computational complexity by quantifying the execution time with increasing sampling frequencies and signal duration.

Finally, we investigate the feasibility of heartbeat detection on functional Near InfraRed Spectroscopy (fNIRS) signals. The technology used for the acquisition of fNIRS signals to study the activation of cortical brain areas is similar to that employed in PPG sensors. Similar to PPG, two light sources (with two different wavelengths) are used to illuminate the scalp, and two detectors measure the amount of transmitted light. The measurements from the two wavelengths are then used to obtain the concentrations of oxygenated and deoxygenated hemoglobin, which are associated with brain activity [13]. Notably, when the fNIRS signals are of good quality [14], it is expected that an oscillatory component derived from blood pulses in the raw signal will be observed.

If isolated from the other signal components, this cardiac component can potentially be used to perform heartbeat detection and estimate the Heart Rate. Given that fNIRS and BVP signals are very similar, we apply the heartbeat detection algorithms designed on BVP signals to detect heartbeats and on fNIRS signals. Our aim is mainly to demonstrate the feasibility and readiness of the approach, and thus motivate future investigations and validation studies.

## 2. DBD, RCO, and MSPTD Algorithms

We briefly review the procedures of the DBD algorithm for the estimation of beat position in BVP signals and the RCO algorithm for error detection and correction. A detailed description can be found in the original paper presenting the two algorithms [12].

### 2.1. The DBD Algorithm

**Figure 1:**
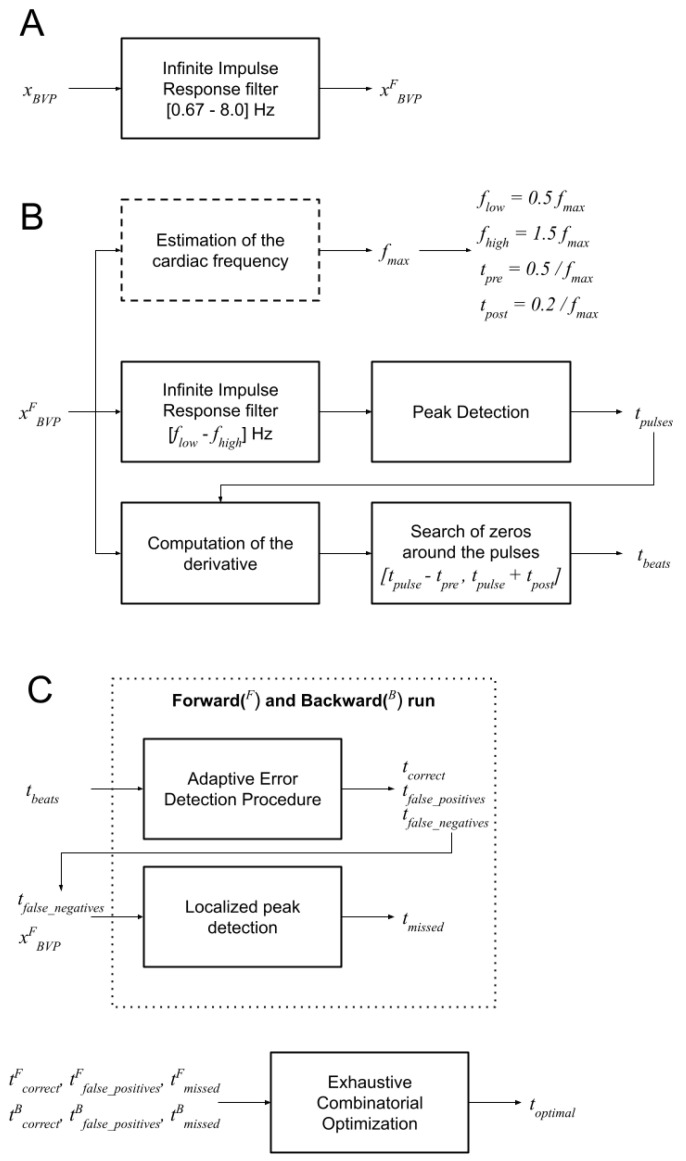
Schematic representation of the DBD and RCO algorithms. (**A**) Signal filtering to remove frequency components not associated with the cardiac activity; (**B**) DBD algorithm. The estimation of the cardiac frequency is optional and used in this study to automatically set the parameters of the DBD algorithm. First, the candidate instants of the heart pulses are estimated on a signal that was filtered to retain only the frequencies around the cardiac frequency. The identified pulse instants are used to center an observation window where the zero of the signal derivative is identified and selected as the heartbeat instant. (**C**) RCO algorithm. Heartbeat detection errors are identified by the Adaptive Error Detection Procedure. Potential missed detections instants (false negatives) are used to identify the target portion for the localized peak detection and obtain candidate heartbeat instants that were missed by the DBD algorithm. The procedure is executed in forward and backward directions. The optimal heartbeat instants are then computed by an exhaustive search for the optimal combination of detected beats from both directions.

**Figure 2:**
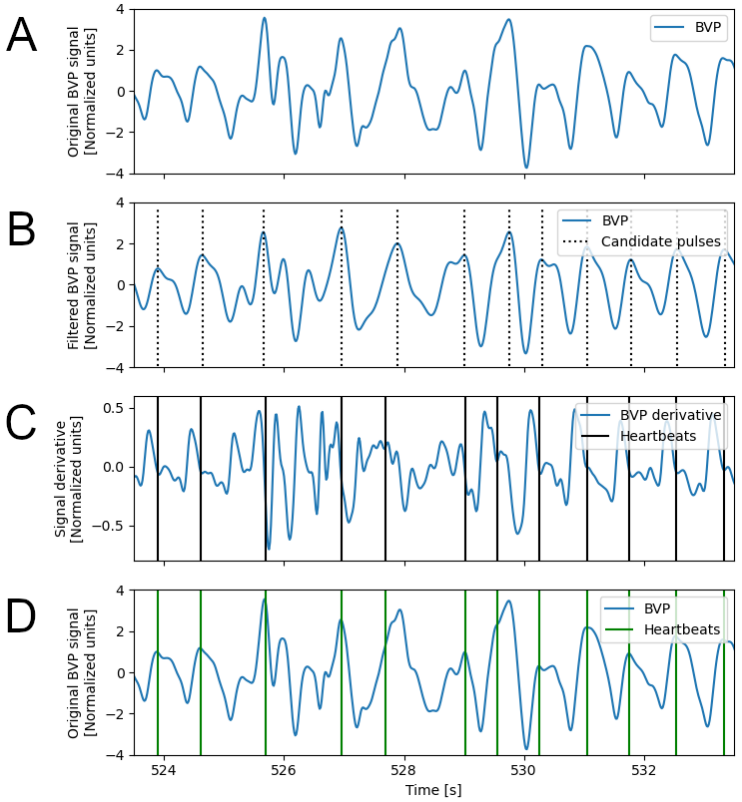
Steps of the DBD algorithm; portion of BVP signal affected by motion artifacts. (**A**) Original BVP signal on which the DBD algorithm is applied; (**B**) Identification of the candidate pulses on the filtered BVP signal; (**C**) Identification of the zeros (or minima) on the derivative of the BVP signal; (**D**) Original BVP signal with the detected heartbeats. Note the effect of the motion artifacts on the heartbeat detection results.

The DBD algorithm (Figure 1 and Figure 2) estimates the position of the percussion peak in BVP signals. The algorithm is regulated by the parameter $f_{\max}$ which is the expected maximal heartbeat frequency. The value of $f_{\max}$ can be empirically set (e.g., to 2 Hz, corresponding to 120 beats per minute), or it can be estimated from the signal’s frequency spectrum (see Figure 1A). In the latter case, the signal is first filtered with a band pass filter (low-frequency cut: 0.67 Hz; high-frequency cut: 8.0 Hz [9]), then the peak frequency of the power spectrum ($f_{peak}$), which is expected to represent the average heart rate, is obtained. The value of $f_{max}$ is then set to $1.5f_{peak}$.

Since we analyzed signals obtained from adults and children of various ages, the value of $f_{max}$ is estimated for each signal from the analysis of the frequency spectrum.

The first step of the DBD algorithm is the application of a band-pass filter (third-order digital Infinite Impulse Response Chebyshev filter, maximum passband ripple: 0.1 dB, minimum attenuation: 40 dB, low-frequency cut: $f_{low}$; high-frequency cut: $f_{high}$); the filtered signal is used to identify the candidate location of the heart pulses, by peak detection (Figure 2B). For each peak instant ($t_{p}$), an interval is defined ($(t_{p}-t_{pre},t_{p}+t_{post}$), which is expected to include the percussion peak, associated with the heartbeat. The instant inside this interval where the signal derivative is minimum (ideally, it should be zero) is considered the heartbeat instant (Figure 2C). Finally, the Inter-beat Intervals (IBI) signal is computed as the sequence of time intervals between the identified heartbeat instants. All parameters of the DBD algorithm can be individually optimized, based on the experimental context and type of application. However, for the sake of simplicity, in this study we opted to link all parameter values to the $f_{max}$ value previously computed: $f_{low}=0.5f_{max}$, $f_{high}=1.5f_{max}$, $t_{pre}=0.5/f_{max}$, $t_{post}=0.2/f_{max}$.

### 2.2. The RCO Algorithm

Noise and MA can affect the local quality of the BVP signal and, consequently, increase the number of detection errors. Among others, two notable errors can occur: (a) a false heartbeat is detected (i.e., false positive), and (b) a true heartbeat is not detected (i.e., false negative).

False positives might affect the possibility of correctly identifying the subsequent true heartbeat. In fact, the interval between the false heartbeat and the true heartbeat might be too short, and the correct heartbeat might be instead detected as an error. Conversely, false negatives cause longer IBI values that, again, could fall outside the acceptable range, and cause the correct heartbeats to be detected as errors.

**Figure 3:**
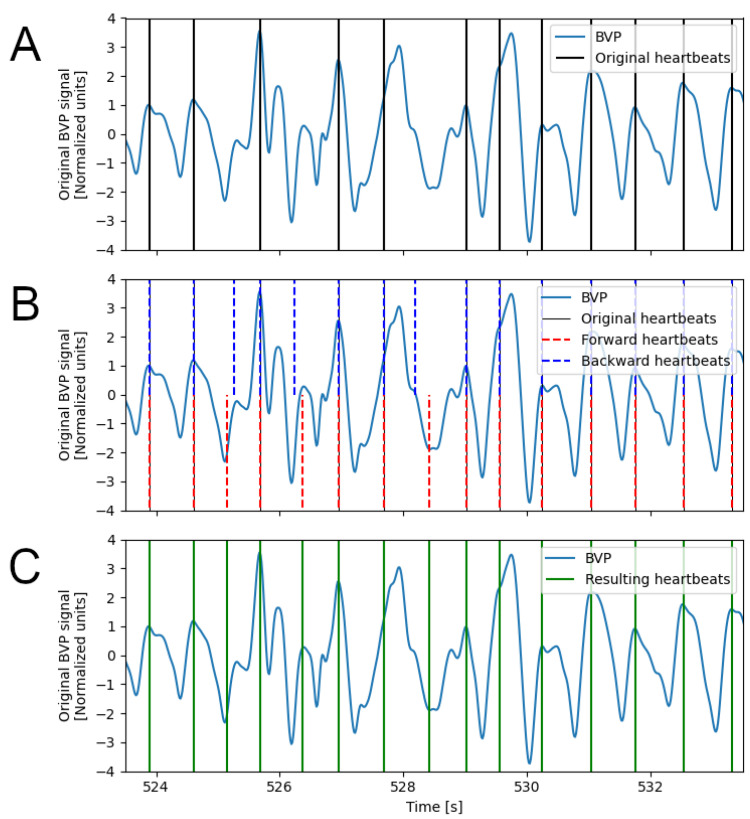
Steps of the RCO algorithm; portion of BVP signal affected by motion artifacts. (**A**) Original BVP signal with the heartbeats detected by the DBD algorithm; (**B**) Results of the AEDP on the forward and backward direction to identify missed peaks and detection errors; (**C**) Results of the RCO with heartbeats selected by the combinatorial optimization of the candidate heartbeats from the AEDP.

The RCO algorithm (Figure 1 and Figure 3) addresses these issues and finds a combination of detected heartbeats that optimizes the variability of the IBI values.

The first step is the identification of heartbeat detection errors, using an Adaptive Error Detection Procedure (AEDP). This is achieved by searching for IBI values that are longer or shorter than the last *k* valid IBI values, given a target range that is computed on the same *k* valid IBI values. The size *k* is empirically set to 3. The target range is based on the median value of the *k* valid values (${I\hat{B}I}_{k}$): [$0.75{I\hat{B}I}_{k},1.25{I\hat{B}I}_{k}$]. The AEDP is applied in both forward and backward directions.

Compared to global approaches to outlier detection (e.g., based on inter-quartiles range), the AEDP allows for adapting detection of the outliers to the local variability of the heart rate, which might change considerably depending on the performed activity.

During the AEDP, new candidate heartbeats are added when an IBI interval is detected as too long, thus indicating a possible missed detection. The candidate heartbeat is selected within the long IBI interval as the instant at which the BVP signal exhibits the highest value and its derivative has the lowest value (Figure 3B). Heartbeats that are identified as false positives in both runs are rejected and those that are detected as true positives in both runs are validated. The remaining beats detected from the backward run are paired with the nearest detected beats from the forward run. For each sequence of consecutive pairs, candidate optimal IBI sequences are composed by exhaustively combining heartbeats from the forward or backward direction. The combination with the lowest standard deviation of the IBI intervals is selected as the optimal combination and the new IBI values replace the original ones (Figure 3C).

### 2.3. The MSPTD Algorithm

The MSPTD algorithm [10] was originally developed to find peaks in intra-cranial pressure signals, based on the “automatic multiscale-based peak detection” [15]. The first step is the computation of the Local Maxima Scalogram (LMS). The LMS is an N×S matrix, where *N* is the number of samples in the signal and *S* is the number of scales, which elements $l_{ij}$ are 1 if the *i*-th sample is greater than the i−j-th and i+j-th samples, and are 0 otherwise. Only the first *d* columns of the LMS matrix are used, where *d* is the scale with highest number of non zero elements. Peaks are then identified as the samples for which the number of non-zero elements (row-wise) is equal to *d*. The procedure is iterated on consecutive overlapping portions of the signal (length: 6 s, overlap: 20%).

## 3. Materials and Methods

The DBD and RCO algorithms have been applied to two datasets: (a) the PPG-DALIA dataset, on which an extensive comparison of existing beat detection algorithms has been performed [9]; and (b) the FANTASIA dataset [16], which provides annotated Blood Pressure signals collected with high-sampling frequency clinical devices. The performances of the DBD and RCO algorithms are compared to the performance of the MSPTD algorithm, which resulted in one of the top-performing algorithms on wearable BVP signals [9].

Subsequently, we focused on the FANTASIA dataset to investigate the role of signal length and sampling frequency on the computational workload of the DBD + RCO, and MSPTD algorithms.

Finally, we demonstrate the feasibility of a heart rate estimation from functional NearInfrared signals, by applying the DBD + RCO and MSPTD algorithms on signals that were processed to represent the cardiac component of fNIRS data.

### 3.1. Datasets

The PPG-DALIA dataset [8], is one of the most important benchmark datasets for heartbeat detection algorithms on BVP signals from wearable data. It includes BVP signals and annotated heartbeat instants from 15 subjects (mean age: 30.6 years) while performing 8 types of every-day activities: sitting, walking, cycling, walking up and down stairs, having lunch, driving, working (at a desk), and playing table tennis. The signals have different durations depending on the type of activity. The BVP signals were acquired with a wearable device (E4, Empatica) with a sampling frequency of 64 Hz. The annotation of the heartbeat instants is based on the Electrocardiogram (ECG) signal collected by a RespiBAN Professional (BiosignalsPLUX, 700 Hz). The signals of the PPG-DALIA dataset are expected to include MA, especially for higher dynamic activities (e.g., playing table tennis), which allows evaluating the robustness and reliability of the algorithms to MA. Contrary to a previous study on the PPG-DALIA dataset, we did not reject bad-quality signals, thus allowing for a more transparent evaluation of the algorithm’s performance in real-world scenarios.

The FANTASIA dataset [16] is one of the few publicly available datasets that contains Blood Pressure (BP) and ECG signals collected with a high-sampling frequency, and annotated heartbeat instants. It was downloaded from PhysioNet [7] and includes data from 20 subjects divided into two age groups: 10 young subjects (mean age: 27 yr) and 10 elderly (mean age = 74 yr). The signals were acquired using a sampling frequency of 250 Hz, while the subjects were relaxed and watching the movie “Fantasia”. In addition to a higher-sampling frequency, compared to the PPG-DALIA dataset, the signals of the FANTASIA dataset are longer and are expected to include less MA, given that the subjects were asked to avoid movement during the signal acquisition experiment. The signal quality is also expected to be better than the PPG-DALIA dataset, as the signals were acquired with a medical-grade device. No signal rejection was performed on the FANTASIA signals either. For the comparison between the DBD + RCO and the MSPTD algorithms, the signals from the FANTASIA dataset were downsampled to 64 Hz, via cubic spline interpolation, to equal the sampling frequency of the PPG-DALIA dataset.

**Figure 4:**
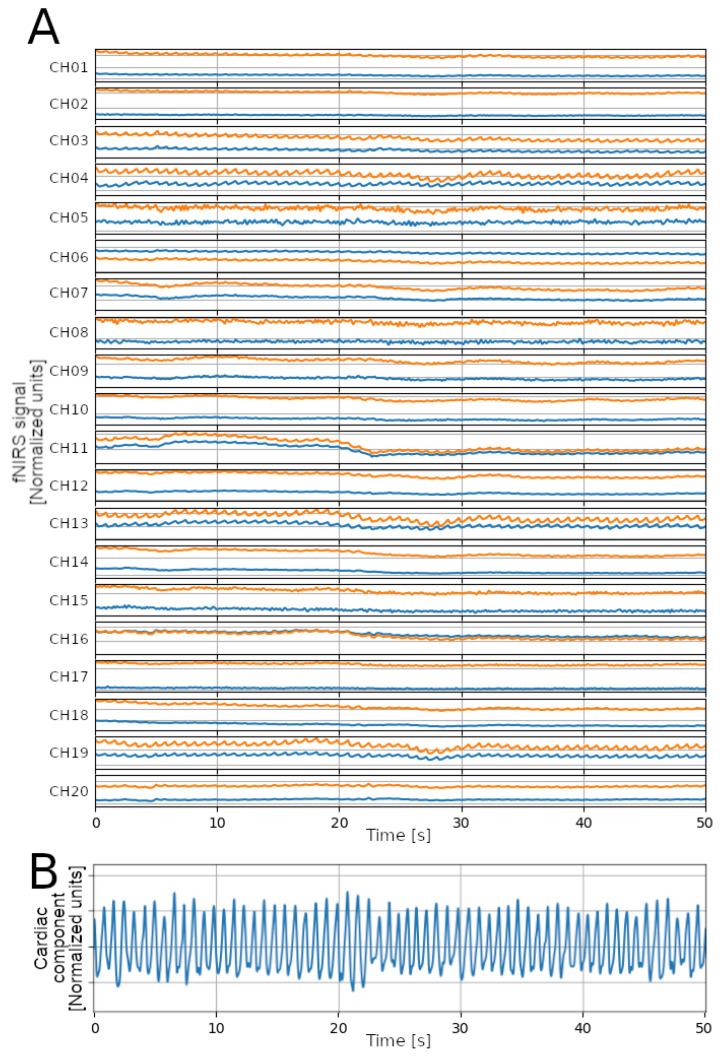
Extraction of the cardiac component from fNIRS data. (**A**) 50 s segment of fNIRS signals for the 20 channels (blue: 760 mn wavelength; orange: 850 nm); (**B**) the obtained signal representing the cardiac component, resulting from the band-pass filtering (0.67–8.0 Hz).

A dataset of parent–child hyperscanning fNIRS data [17] was used to evaluate the feasibility of the heart rate estimation on fNIRS data. This dataset has been used to study the synchrony between parents and children during shared video watching and play [18,19,20,21]. It was selected among others because: (a) it includes subjects from two age groups: adults and children; and (b) it collects data during two experimental conditions: while the subjects were watching some videos (thus, few MA are expected), and while the subjects were playing (more MA expected). The fNIRS signals were acquired using a NIRSport device (7.81 Hz, NIRx Medical Technologies) and processed to obtain one signal for each subject, representing the cardiac component of the fNIRS signals (see Figure 4). Specifically, signals were upsampled to 64 Hz via cubic spline interpolation; then, for each sampling instant, the values from each channel (20 channels) and wavelength (2 wavelengths: 760 nm and 850 nm) were averaged. After this step, the obtained signal is considered a BVP signal; as such, the detection of heartbeats is performed as with the signals from the PPG-DALIA and the FANTASIA datasets.

Except for those described in the previous paragraphs, no other pre-processing step was performed on the signals prior to the application of the heartbeat detection algorithms.

### 3.2. Analysis

This study had three aims: (a) Comparing the performances of the DBD, DBD + RCO, and MSPTD algorithms; (b) evaluating the impact of the signal-sampling frequency and duration on the computational workload; and (c) demonstrating the feasibility of local heart rate estimation from fNIRS signals.

Similarly to what was performed in [9], the comparison of the heartbeat detection results with the different algorithms is based on the reference heartbeat annotations, starting from the count of correctly detected beats (true positives), erroneously detected beats (false positives), and missed detections (false negatives). First, we corrected the offset between the detected and reference heartbeats. The reference heartbeats are typically obtained from other signals than the BVP signal used for the heartbeat detection (e.g., from the ECG); thus, the heartbeat pattern on different signals (e.g., R-peak in ECG, percussion peak in the BVP) might occur with an offset caused by blood flow dynamics, anatomical distance, or technical settings (e.g., using different and independent acquisition systems). Since the detection of true and false positives is based on the temporal distance between reference and detected heartbeats, it is critical to estimate and correct the offset to avoid external factors influencing the evaluation of the algorithm performance. The offset is estimated by bootstrapping the cross-correlation between the detected and reference heartbeat series.

First, a 30 s interval is randomly selected and used to extract the two corresponding portions from the detected and reference heartbeat series. The IBI values of the two portions are computed and interpolated (cubic spline interpolation, 100 Hz), and the cross-correlation is obtained. Since the interpolation frequency was 100 Hz, the increment between the lags was 1/100 Hz = 0.01 s, thus the resolution to determine the lag was 0.01 s. The lag corresponding to the maximum cross-correlation value is considered the offset between the detected and reference heartbeats. The procedure is performed 5000 times, each time on a different, randomly selected, portion, thus obtaining a distribution of estimated offset values: the median of such distribution is obtained and used to correct the offset between the reference and detected heartbeat series. The correction of the offset is performed subject-wise and for each heartbeat detection algorithm.

We then paired each detected heartbeat to a heartbeat from the reference series. The pairing is considered valid when the distance between the detected and reference beat is below 150 ms. The number of successfully paired beats is the number of True Positives (TP); the number of unpaired detected beats is the number of False Positives (FP); and the number of unpaired reference beats is the number of False Negatives (FN). Finally, we computed the overall Sensitivity ($Sens=100\frac{TP}{TP+FN}$), Positive Predictive Value ($PPV=100\frac{TP}{TP+FP}$), and F1 score ($F1=\frac{2*PPV*Sens}{PPV+Sens}$). A One-way Analysis of Variance (ANOVA) was performed on the F1 scores to assess whether the different algorithms achieve a statistically different performance. The ANOVA is performed for each session of the PPG-DALIA dataset and on the FANTASIA dataset; post hoc pair-wise *t*-tests were performed on significant results. The Bonferroni correction was applied to correct the statistical significance threshold for the multiple hypotheses; specifically, since we ran 9 different tests (8 sessions of the PPG-DALIA dataset and the FANTASIA dataset), the significance threshold for the one-way ANOVA was set to α=0.05/9=0.006.

To evaluate the impact of the sampling frequency and signal duration on the computational workload of the different algorithms, we referred to the FANTASIA dataset only. To estimate the computational workload, we considered the wall time required to perform the different algorithms, specifically: the DBD, the RCO, and the MSPTD. The algorithms were applied on segments of signals with five different durations (10 s, 60 s, 300 s, 900 s, and 1800 s) and three different sampling frequencies (64 Hz, 128 Hz, 250 Hz). All segments started after the first 10 s of the signal.

For the feasibility study of a local heart rate estimation based on fNIRS signals, no annotated heartbeats were available to allow for an evaluation of the algorithm’s performance and an assessment based on qualitative indications. We report general statistics of the estimated heart rate series, with a comparison of the results from the DBD + RCO and MSPTD procedures. In addition, we apply the AEDP on the forward direction and report the number of detection errors.

All computations were performed on a Lenovo ThinkCentre workstation (CPU: AMD Ryzen 5 PRO 3400G; Memory: 16 GB; OS: Ubuntu 22.04.1 LTS; Python: 3.10.8), based on the *pyphysio* package [22].

## 4. Results

We compared the detection performance of the algorithms in terms of capability to detect the true heartbeats, computational workload, and its dependence on the sampling frequency and length of the signal. Finally, we considered the results of the application of the same algorithms on the cardiac component derived from fNIRS data.

### 4.1. Comparison of DBD, DBD + RCO, and MSPTD

**Figure 5:**
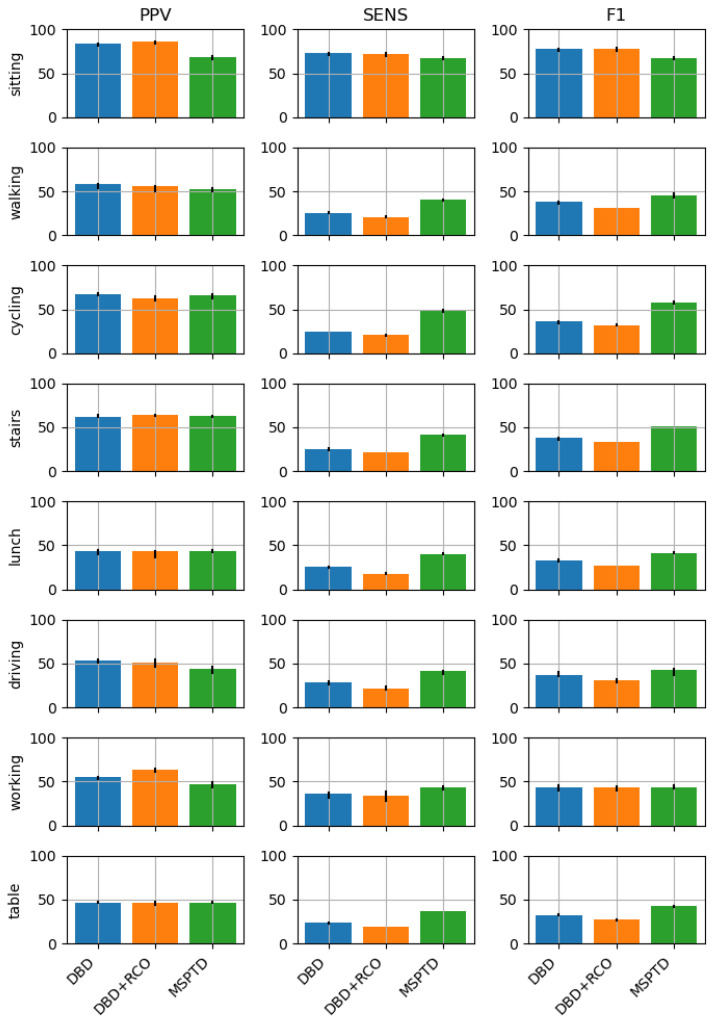
Performances of the heartbeat detection algorithms on the PPG-DALIA dataset.

**Figure 6:**
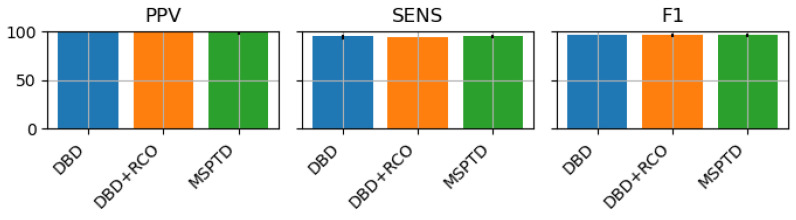
Performances of the heartbeat detection algorithms on the FANTASIA dataset.

We used the three algorithms considered in this study to detect heartbeats from two datasets: PPG-DALIA (signals collected with wearable devices, low-sampling frequency, and during different activities), and FANTASIA (signals collected with medical-grade devices, high-sampling frequency, resting state). We compared the results in terms of Sensitivity (Sens), Positive Predicted Value (PPV), and F1 score (F1). The performance on the PPG-DALIA and the FANTASIA datasets is reported in Figure 5 and Figure 6, respectively; Table (Table 1) reports the statistics of the F1 score on the PPG-DALIA (by activity) and FANTASIA datasets, for the different algorithms, and the results of the one-way ANOVA tests.

The results from the one-way ANOVA tests (Table 1) showed significant performance differences between the algorithms for the *cycling* (F(2, 39) = 6.38, *p* = 0.004), and the *stairs* (F(2, 33) = 6.16, *p* = 0.005) activities only. In both cases, the MSPTD algorithm achieved a statistically better performance than DBD (*cycling*: t(26) = 2.98, *p* = 0.006; *stairs*: t(22) = 2.51, *p* = 0.020) and DBD + RCO (*cycling*: t(26) = 3.48, *p* = 0.002; *stairs*: t(22) = 3.32, *p* = 0.003), while no statistical difference was found between DBD and DBD + RCO.

Considering the mean values of the performance scores on PPG-DALIA, we observe that the performances of the DBD and DBD + RCO are superior or equal to the performance of the MSPTD in low-dynamic activities (sitting and working). In all the other activities, although the three algorithms are comparable in terms of PPV (with slightly better performances for DBD or DBD + RCO), MSPTD is superior in terms of Sensitivity, resulting also in a higher F1 score. Results on FANTASIA, which include resting-state data, confirm the findings on PPG-DALIA: the performances of the three algorithms are comparable. Additionally, we note that compared to results from the sitting activity of the PPG-DALIA, the performance is much higher.

### 4.2. Computational Workload

**Figure 7:**
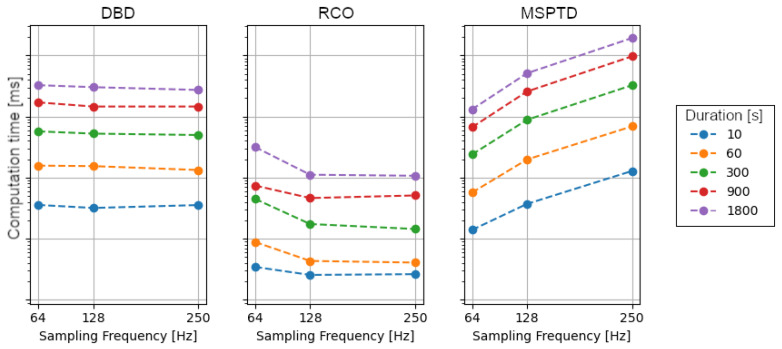
Computation times of the heartbeat detection algorithms for increasing sampling frequency and duration of the signals.

Based on the FANTASIA dataset, we compared the computation time of the three algorithms with signals of different lengths and sampling frequencies, as a measure of the computational workload (Figure 7). Given a fixed sampling frequency, the computation times of all three algorithms are positively associated with the signal lengths. For instance, at a sampling frequency of 128 Hz, the computation times of DBD, RCO, and MSPTD increase by 16.5 ms/s, 0.6 ms/s, and 28.4 ms/s, respectively. Given a fixed signal length, only the computation time of the MSPTD appears to depend on the sampling frequency, with exponential growth. We also note a drop in the computation time for the RCO algorithm, from 64 Hz to 128 Hz, which is probably associated with a reduced number of beat detection errors.

### 4.3. Application to fNIRS Data

**Figure 8:**
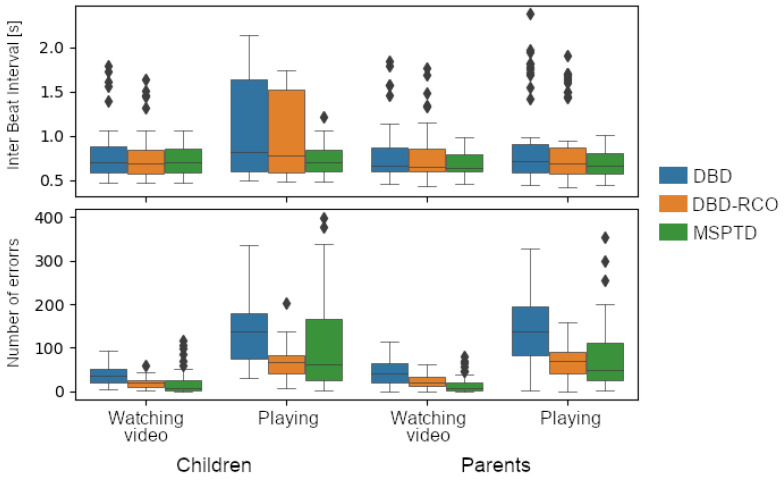
Results of the application of the heartbeat detection algorithms on the cardiac component signal derived from fNIRS data. (**Top**) distribution of the average InterBeat Interval length; (**Bottom**) distribution of the number of detection errors.

The three algorithms were applied on the cardiac component signals derived from fNIRS data. No ground truth was available, so a comparison in terms of performance, as done on the PPG-DALIA and FANTASIA datasets, was not feasible. However, we qualitatively compared the results in terms of ranges of the average IBI duration, and in terms of errors detected by the AEDP (Figure 8). Except for the play session for children, the IBI duration ranges were comparable across the three algorithms, and in the physiological range. According to the AEDP, the MSPTD algorithm produced fewer detection errors. Again, the number of errors is lower for the video session, which is expected to include less MA.

## 5. Discussion

In this paper, we analyzed three algorithms for heartbeat detection from BVP signals. In particular, we compared the DBD and the RCO algorithms with a state-of-the-art algorithm: the MSPTD algorithm. These DBD and RCO algorithms were designed to be mostly applied on signals from wearable devices [12]. Therefore, we evaluated them on the PPG-DALIA dataset [8], composed of signals acquired with wearable devices. Most importantly, this study builds on the work of Charlton and colleagues [9], who reviewed, compared, and assessed, the performance of the most adopted heartbeat detection algorithms for BVP signals. According to their study, the MSPTD algorithm achieved the highest performance on multiple datasets. By considering the MSPTD algorithm as the reference algorithm, we intended to compare the performances of the DBD and RCO algorithms with the state-of-the-art results in the field.

However, in this study we also considered poor-quality signals, and signals from a dataset that used medical-grade devices—the FANTASIA dataset [16]. Since signals from the FANTASIA dataset were collected with a higher-sampling frequency, we could also evaluate the computational workload required to run these algorithms, and its dependence on the length and sampling frequency of the signal.

The results indicate that the performances of the three algorithms are comparable when the signals are collected during activities with limited movements (e.g., sitting, working, resting), while the MSPTD is superior in the case of activities that include high-magnitude body movements (cycling and walking up and down stairs). The superiority is mainly due to better sensitivity.

The sitting session of the PPG-DALIA dataset and the FANTASIA dataset were both collected while subjects were in a resting state; however, we observed better performance scores on the FANTASIA dataset. We postulate that this is mainly due to the higher-quality signals collected by the medical-grade device used in the FANTASIA dataset, and to the fact that resting supine, compared to sitting, was probably more effective at limiting body movements. Instead, the higher-sampling frequency used for the FANTASIA dataset should not play any role, since the signals were downsampled to 64 Hz (sampling frequency of the PPG-DALIA dataset) before the beat detection procedure.

Notably, in our analyses, the performance obtained by the MSPTD on the PPG-DALIA dataset was lower than that reported in [9]. For instance, for the *sitting* activity, which resulted as the one with the best performance in both studies, Charlton and collegues reported a median F1 score of 95.1, while we found a mean score of 83.6. When the subject was playing table soccer (*table* activity), we reported a mean F1 score of 54.3, while Charlton and colleagues reported a median F1 score of 61.0, the worst performance across all activities. Since the implementation of the MSPTD algorithm was the same in both cases, we can conclude that this difference is mainly due to the fact that in our study we omitted the data selection step, where signals of low quality or the presence of severe MA are discarded. These results confirm the importance of extending the assessment of the heartbeat detection performance on signals collected in real-world contexts; while the findings of Charlton and colleagues provide a valuable indication of the maximal performance that can be expected, our results will be useful to researchers that work with real-life applications, where the actual quality of the input data cannot be controlled.

Furthermore, we considered the computational workload required by each algorithm, estimated in terms of computation time. While for all algorithms the computation time was positively associated with the signal duration, we observed a sensitive dependence on the sampling frequency only for the MSPTD algorithm. In particular, the computation time appeared to increase exponentially with the sampling frequency.

These results suggest that the use of the DBD and DBD + RCO algorithms could be more appropriate in clinical contexts, where the subjects are expected to avoid body movements and medical-grade devices with high-sampling frequency are used to collect the signals. However, the aim of the heartbeat detection task should also be considered: the DBD and DBD + RCO algorithms could perform better when a lower number of false heartbeats is required, at the expense of a higher number of missed heartbeats. While low-sampling frequency signals are more than appropriate for the estimation of the Heart Rate, which is typically reported in beats per minute, a higher-sampling frequency would be required for the analysis of Heart Rate Variability, where the IBI intervals should be quantified with a resolution of 1 ms.

Finally, we tested the feasibility of heartbeat detection on the cardiac component obtained from fNIRS data derived from a public fNIRS dataset [17]. This approach could enable the observation of heart rate variations across the experiment, in parallel with the quantification of brain activity. Additional research that includes a rigorous assessment of the reliability of such estimates is needed, also considering the low original sampling frequency (e.g., 7.81 Hz in the dataset used in this study). In our study, we did not validate the heartbeat detection algorithms by a comparison with ground truth information, which is needed to provide researchers with an indication of the reliability of the results. However, we demonstrated that with straightforward signal-processing steps, the cardiac component can be extracted from fNIRS data and used for a heartbeat detection procedure, similar to any other BVP signal.

## 6. Conclusions

In this study, we compare three algorithms for heartbeat detection: the MSPTD algorithm, which, according to an extensive review by Charlton and colleagues [9], is one the most accurate algorithm reported in the literature; and the DBD and RCO algorithms, that, although introduced in a previous study [12], have never been compared on benchmark datasets with other algorithms. The comparison was based on the PPG-DALIA dataset, which included signals collected via wearable devices while the subjects were performing nine different activities, and on the FANTASIA dataset, composed of signals collected using medical-grade devices while the subjects were resting supine.

Except for the activities in the PPG-DALIA that involved high-magnitude body movements (namely: cycling and walking up and down stairs), the three algorithms achieved a similar performance across different types of activities and devices used for the signal acquisition. This confirms that the DBD and RCO algorithms are capable of achieving a heartbeat detection performance in line with the MSPTD, one of the state-of-the-art algorithms. Contrary to the reference study, which focused on high-quality signals only [9], we avoided any preferential selection of signals based on signal quality. Consequently, the obtained performance scores were lower, but also closer to the performance that can be expected in real-life applications. Finally, we also considered the computational complexity of the algorithms and found that the computational time required by the MSPTD algorithm to operate grows exponentially with the sampling frequency of the signal. These findings extend those reported by Charlton and colleagues [9], providing additional indications that researchers should consider in the selection of the most appropriate algorithm for heartbeat detection on BVP signals.

Finally, we demonstrated that from a signal-processing perspective, the investigated heartbeat detection algorithms might be appropriate to process the cardiac component of fNIRS data and obtain a local indication of the heart rate. However, additional investigations are needed to ensure the approach is reliable and robust by comparing the results with a reference ground truth, a consideration that was not included in this study.

## Figures and Tables

**Table 1:** Statistics of the F1 score for the different activities of the PPG-DALIA dataset, and on the FANTASIA dataset, for the different algorithms, and results of the one-way ANOVA to test the performance differences between the algorithms (α=0.006).

	DBD	DBD + RCO	MSPTD	
	Mean (SD)	Mean (SD)	Mean (SD)	One-Way ANOVA
PPG-DALIA Session				
sitting	89.7 (7.6)	89.6 (7.6)	83.6 (11.1)	F(2, 42) = 2.31; *p* = 0.112
walking	56.8 (16.2)	53.6 (18.9)	62.9 (12.1)	F(2, 36) = 1.15; *p* = 0.328
cycling	54.4 (21.0)	49.5 (22.9)	74.7 (14.4)	F(2, 39) = 6.38; *p* = 0.004
stairs	50.5 (11.9)	45.0 (14.0)	62.9 (12.2)	F(2, 33) = 6.16; *p* = 0.005
lunch	48.4 (14.0)	43.0 (15.8)	53.7 (10.9)	F(2, 33) = 1.85; *p* = 0.173
driving	67.4 (19.4)	65.5 (22.4)	67.0 (18.0)	F(2, 42) = 0.04; *p* = 0.962
working	69.7 (18.9)	70.6 (18.3)	64.0 (14.3)	F(2, 39) = 0.60; *p* = 0.553
table	41.4 (13.8)	37.5 (17.0)	54.3 (12.8)	F(2, 36) = 4.70; *p* = 0.015
FANTASIA	99.2 (1.3)	99.1 (1.5)	99.2 (1.3)	F(2, 57) = 0.09; *p* = 0.914

## Data Availability

Publicly available datasets were analyzed in this study. This data can be found here: PPG-DALIA: https://archive.ics.uci.edu/ml/datasets/PPG-DaLiA, accessed on 21 March 2023; FANTASIA: https://physionet.org/content/fantasia/1.0.0/, accessed on 21 March 2023.
